# Mucosa-Associated Lymphoid Tissue Lymphoma of the Sublingual Salivary Gland: A Case Report

**DOI:** 10.7759/cureus.38179

**Published:** 2023-04-26

**Authors:** Monika Aleksiejūnaitė, Andrius Talijūnas, Linas Zaleckas, Rūta Rastenienė

**Affiliations:** 1 Centre of Oral and Maxillofacial Surgery, Faculty of Medicine, Vilnius University Hospital Žalgiris Clinic, Vilnius, LTU

**Keywords:** sublingual gland lymphoma, malt lymphoma, salivary gland, b-cell lymphoma, non-hodgkin’s lymphoma

## Abstract

Mucosa-associated lymphoid tissue (MALT) lymphoma is a type of B-cell lineage lymphoma that can affect the head and neck region. This report presents a rare case of an extra-nodal marginal zone B-cell MALT lymphoma of a sublingual gland, diagnosed in an 18-year-old male patient. The patient had a history of ranula surgical removal on the right side of the mouth. One year after surgery, the patient presented with complaints of swelling of the left parotid gland, with no significant changes found during the examination and a self-resolving outcome. Subsequently, two years later, the patient began to complain of a fast-growing cyst under the tongue. A surgical excision of the left sublingual gland and the ranula was performed, and a final diagnosis of MALT lymphoma was rendered. The patient was referred to the department of hematology for further treatment planning and follow-up.

## Introduction

Lymphomas are a type of hematolymphoid neoplasms characterized by the proliferation of malignant lymphoid cells. These neoplasms can be classified into two primary groups: Hodgkin lymphomas and non-Hodgkin lymphomas (NHLs) [1]. NHLs are a part of a heterogeneous group of malignant lymphocytic neoplasms whose prevalence has recently been increasing [2]. Primary oral cavity non-Hodgkin lymphoma is very rare and manifests in approximately 3%-5% of all NHLs [3,4]. Oral mucosa-associated lymphoid tissue (MALT) lymphoma is frequent in males, with the age ranging from 3 to 96 years [1]. The most affected anatomical regions are the stomach (30%), orbit (12%), skin (10%), and lungs (9%) [5]. Lymphomas of the salivary glands represent around 1.7%-3.1% of all salivary gland neoplasms and the incidence is 79% in the parotid glands, 18% in the submandibular glands, 2% in the minor salivary glands, and 1% in the sublingual glands [4,6].

MALT lymphoma is a non-Hodgkin lymphoma subtype and is an indolent B-cell neoplasm virtually always presenting in extra-nodal sites [2,3,7]. Risk factors for the MALT lymphomas are periodic swellings of the parotid gland, increased mononuclear cell infiltrates in the lower lip biopsy, and positive rheumatoid factors [8]. The current report describes a rare case of MALT lymphoma of the sublingual salivary gland, after the removal of the ranula in an 18-year-old patient.

## Case presentation

An 18-year-old male patient presented to the Vilnius University Hospital Žalgiris Clinic with a fast-growing cyst under his tongue, with the complaints starting two weeks ago. A ranula surgical removal done four years ago and the swelling of the left parotid gland three years ago with a successful self-resolving outcome were recorded in the prior medical history.

**Figure 1:**
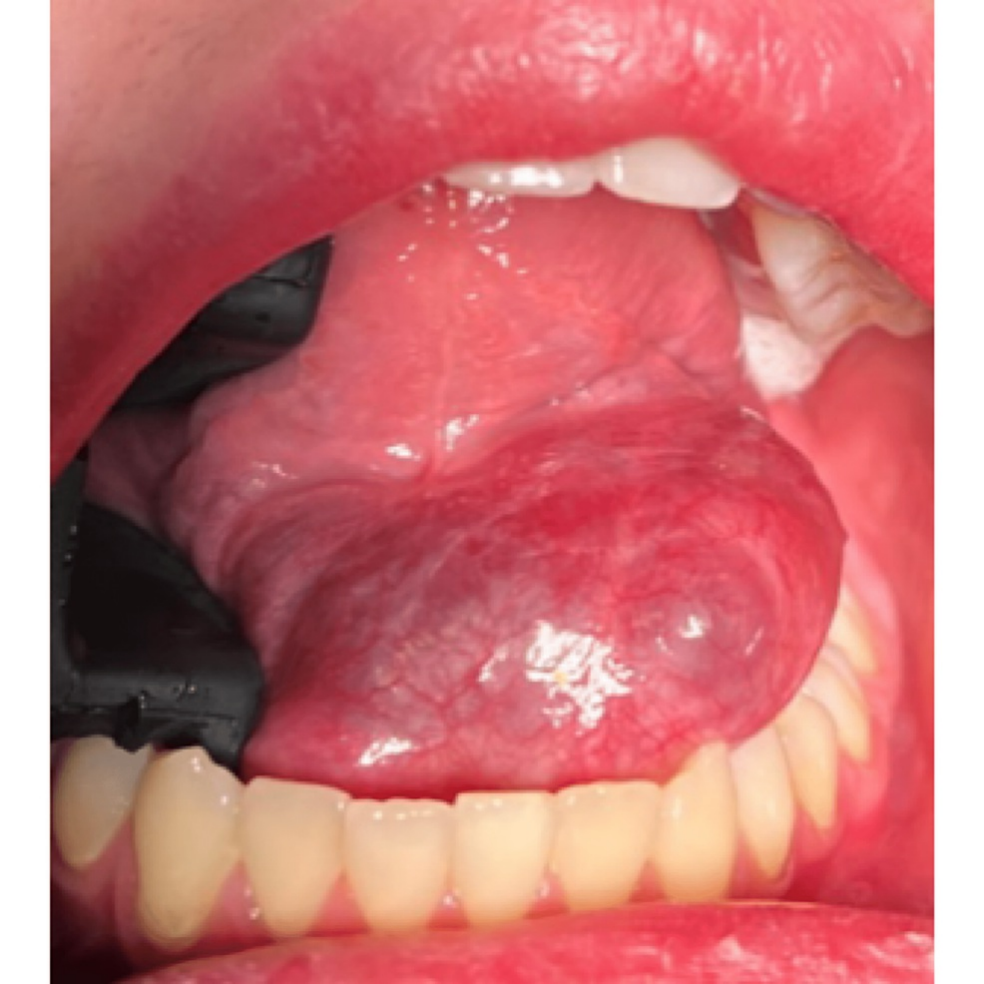
Intraoral view of the cystic lesion in the left sublingual region

During an intraoral examination, a painless, smooth surface, thin-walled, fluctuant cyst with around 35-mm diameter and mucosa-like color was observed on the left side of the floor of the mouth (Figure 1).

There was no enlargement of any salivary glands; no lymph nodes were palpated in the head and neck region. Ultrasound scans showed an inhomogeneous, space-occupying 37 x 23 mm cystic lesion on the floor of the mouth and a view of generalized sialadenitis of the left parotid gland with no tumors. Sjogren’s syndrome (SS) was suspected after the examination.

**Figure 2:**
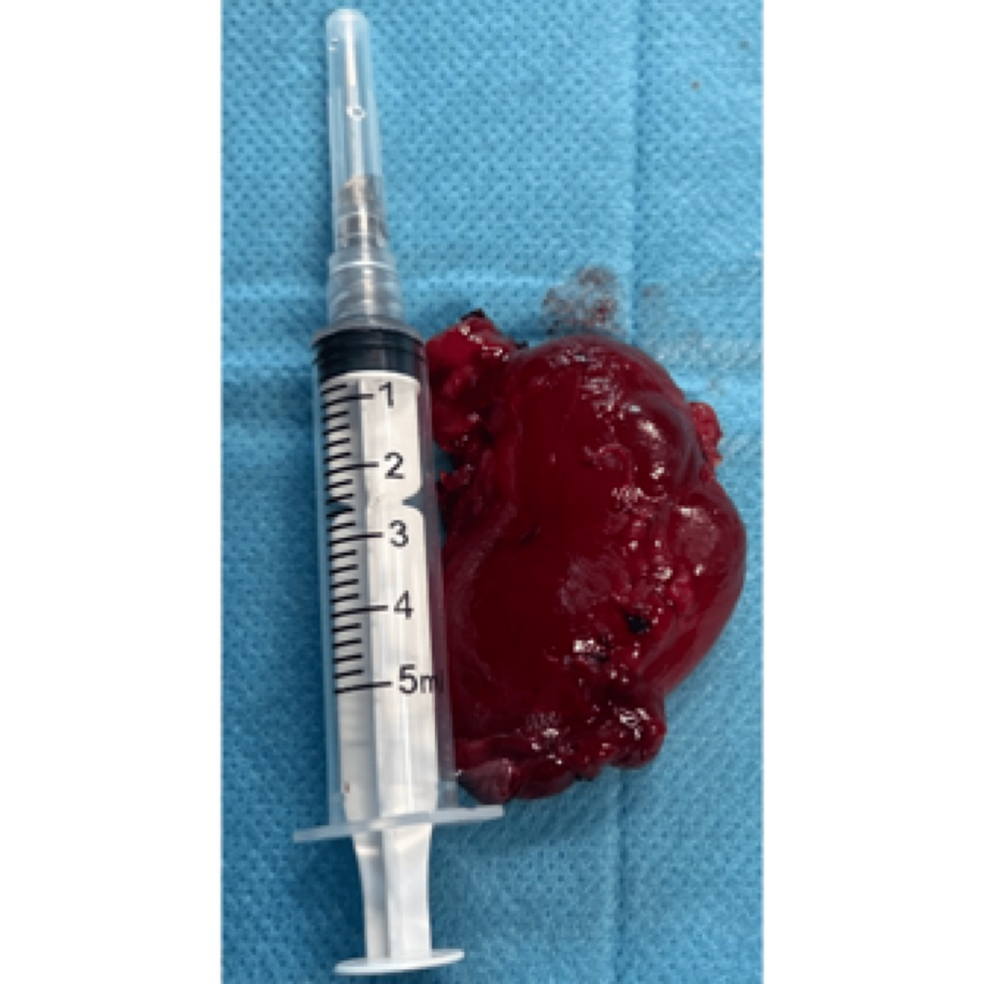
Ranula after the removal

**Figure 3:**
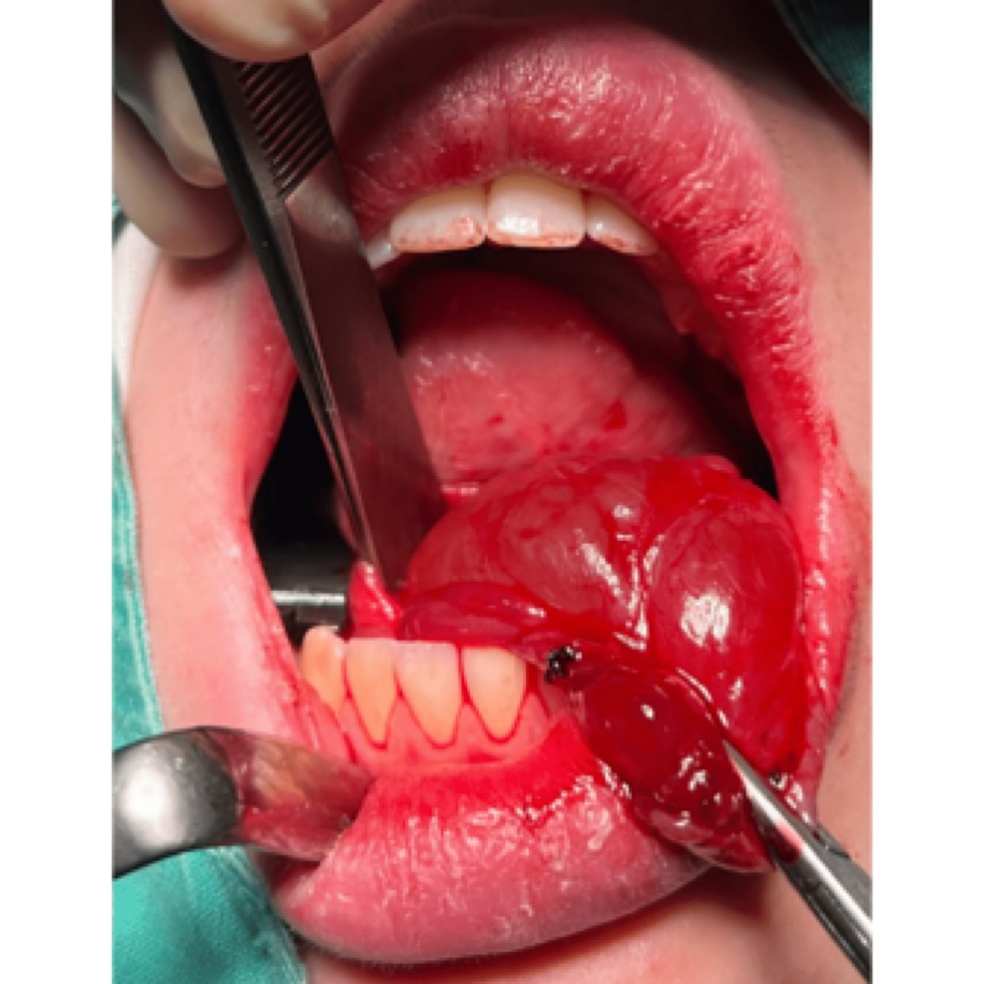
Surgical excision of the ranula and sublingual salivary gland

**Figure 4:**
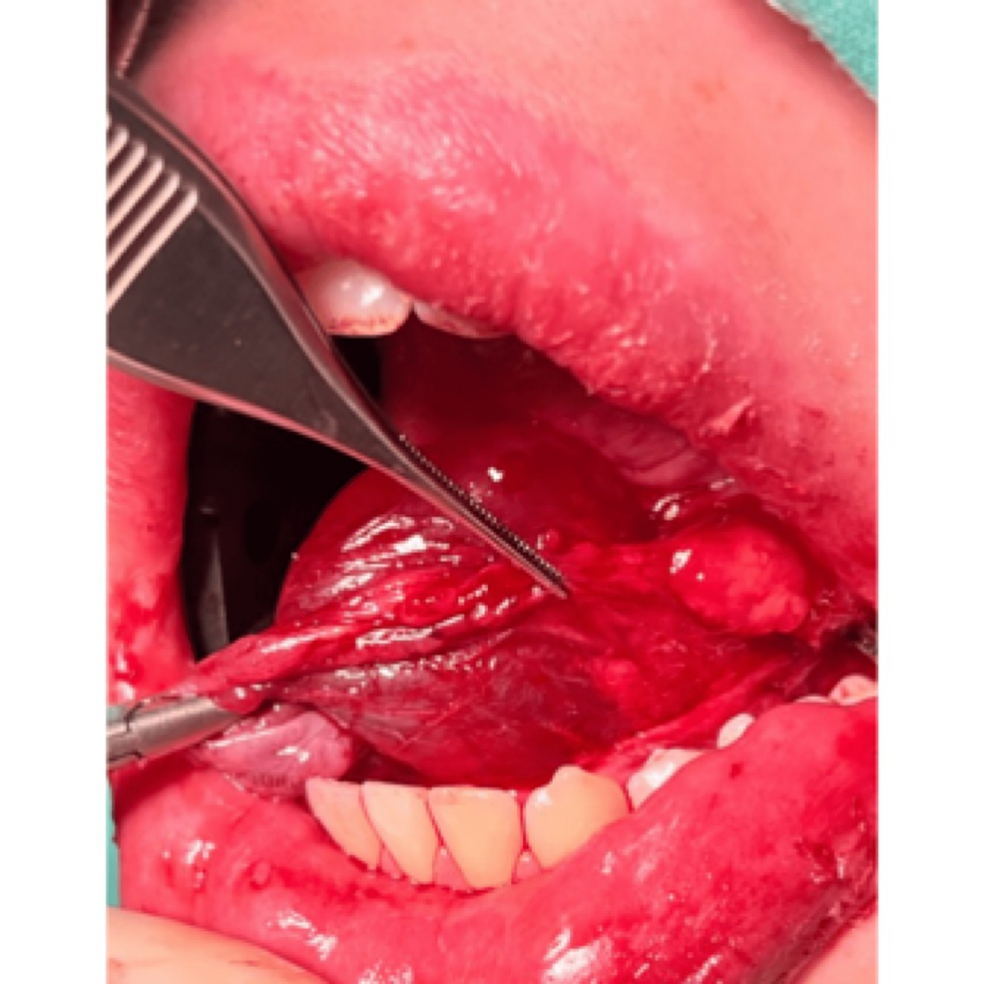
Surgical excision of the ranula and sublingual salivary gland

**Figure 5:**
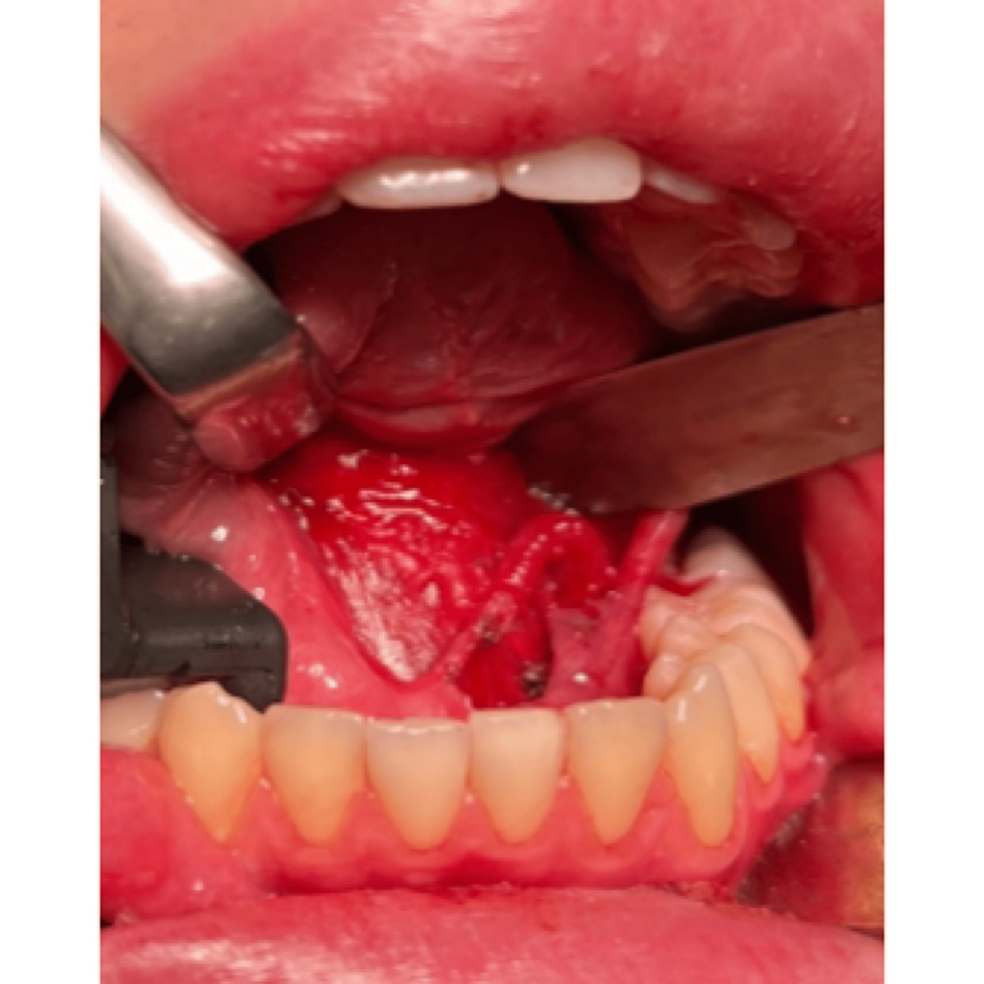
Surgical excision of the ranula and sublingual salivary gland

**Figure 6:**
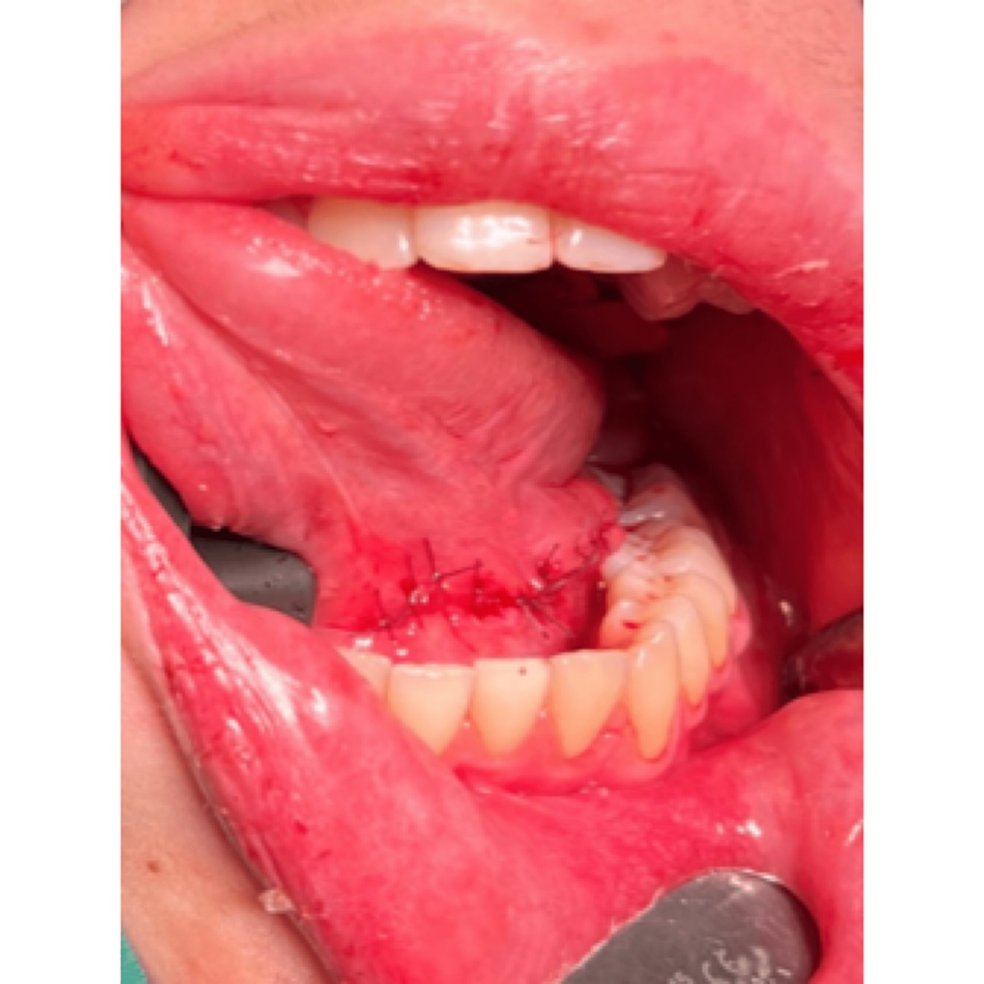
Surgical excision of the ranula and sublingual salivary gland

A complete surgical excision of the ranula and removal of the left sublingual gland were performed under general anesthesia (Figures 2-6).

A histopathological investigation revealed an extra-nodal marginal zone B-cell MALT lymphoma and ranula. The patient was referred to the department of hematology. Following diagnosis, the patient underwent additional diagnostic examinations, including bone marrow aspiration and biopsy conducted by the department of hematology. These tests showed evidence of reactive bone marrow processes; components of the complete blood count as well as lactate dehydrogenase levels were within the normal range. An abdominal and pelvic ultrasound examination was also performed that did not reveal any enlarged lymph nodes that could be attributed to the disease. Based on the results of these tests, the patient was diagnosed with regional non-Hodgkin’s IA stage disease, according to the WHO classification. Consistent with treatment protocols in Lithuania, the patient, being young and without B symptoms or splenic involvement, underwent complete surgical excision without requiring further treatment. Follow-up appointments were scheduled every five months for monitoring purposes and after the additional examination, no further treatment was required.

**Figure 7:**
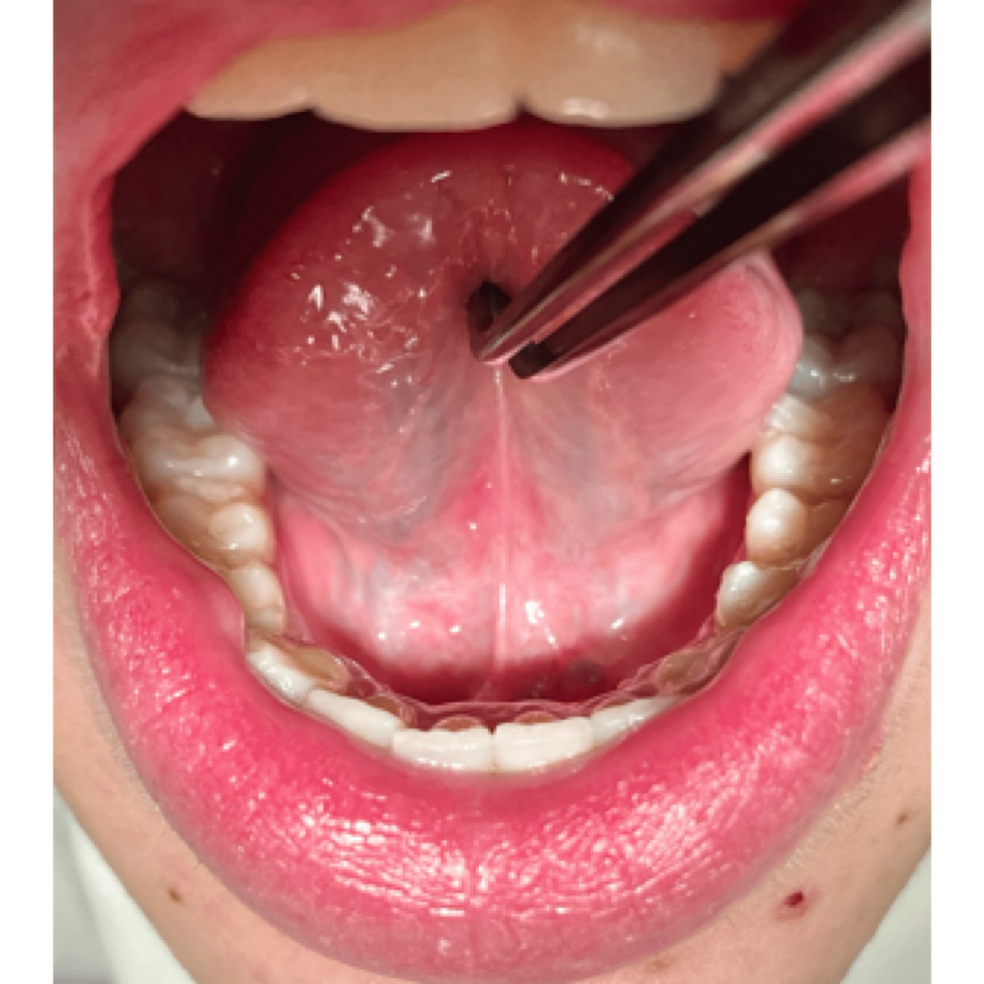
Sublingual site during the follow-up examination

During the first follow-up visit after six months, no significant progression of the disease was noticed (Figure 7).

One year after the surgical treatment, the patient came with an enlargement of a labial salivary gland. Few enlarged labial salivary glands were observed during a clinical examination; an ultrasound scan showed a pathological change in a cervical lymph node. The biopsy of the labial salivary gland revealed focal lymphocytic sialadenitis with symptoms characteristic of Sjogren's syndrome. The diagnosis of SS was confirmed by a rheumatologist based on clinical presentation, serological tests, and salivary gland biopsy results.

## Discussion

The two most common histological subtypes of NHL are a clinically aggressive diffuse large B-cell lymphoma and MALT lymphoma. Mucosa-associated lymphoid tissue lymphoma is an indolent B-cell neoplasm virtually always presenting in extra-nodal sites [3,7]. MALT lymphomas have unique clinical features and histological morphology forming lymphoepithelial lesions [9].

MALT lymphomas in the salivary gland are low-grade indolent malignancies that require long‐term patient observation. Practice guidelines for B-cell lymphomas recommend a follow-up for five years [10]. However, MALT lymphomas can turn into aggressive diffuse large B-cell lymphomas and the survival rate decreases sharply [5,11]. Moreover, salivary gland tissues normally do not involve lymphocytes and contain them during inflammation [12].

Chronic infection, inflammation, autoimmune disorders, advancing age and genetics can be risk factors for the development of MALT lymphomas [13]. Only 5% of patients with autoimmune diseases are diagnosed with non-Hodgkin lymphomas. Primary Sjogren’s syndrome patients are at a higher risk of lymphoma than patients with other autoimmune diseases [14,15]. Compared with other extra-nodal lymphomas, MALT lymphoma is always associated with SS and manifests slowly in clinical presentation [9,14]. Sjogren’s syndrome is a systemic autoimmune disease related to an increased risk of lymphoma, as SS leads to oral and ocular dryness, that may cause local complications in the mucosa [11,14]. The clinical picture of Sjogren’s syndrome combines symptoms of dryness of the mucosa in many parts of the body, fatigue, and arthralgia in almost all patients [11]. MALT lymphomas affect salivary glands in the context of lymphoid tissue hyperplasia associated with chronic inflammatory conditions [12,13,15]. Diet, smoking, alcohol consumption, genetic predisposition, occupational and environmental factors are also of high importance in the development of lymphomas [16].

Treatment options for MALT lymphomas depend on the presence of localized or disseminated disease and are based on the International Prognostic Index score parameters: patients' age, stage of the disease, involvement of extra-nodal sites, performance status, and lactate dehydrogenase range [17]. High mortality is noticed in patients over 60 years of age; a better survival rate is seen in patients at disease stage 1. There is a lower mortality risk in patients with lower disease stage and performance status >2 [17,18]. The prognosis of lymphomas involving the salivary glands depends on the pathological subtypes and staging. In general, patients with MALT and follicular lymphomas have a better prognosis than patients with diffuse large B-cell lymphomas [16].

The treatment of sublingual gland lymphomas includes surgery, chemotherapy and radiotherapy depending on the lymphoma subtype [10]. According to Hussein, in a series of 37 patients with oral lymphomas, radiation therapy was successful in limited cases whereas high-grade lymphomas required combined chemotherapy and radiotherapy [1].

Fu et al. found that for head and neck MALT lymphomas, surgical treatment with or without chemoradiotherapy had no significant difference when it came to overall survival. Complete resection of the tumor during the surgery can achieve therapeutic effects and reduce the adverse reactions and economic burden caused by chemoradiotherapy [9]. In patients with localized low-grade lymphomas affecting the exocrine glands, surgery alone can be an option [4,9,11]. Moreover, chemoradiotherapy can cause and aggravate the symptoms of xerostomia, causing deterioration of oral hygiene, swallowing function, taste, and voice, forming a vicious cycle [9].

## Conclusions

Sublingual gland's extra-nodal marginal zone B-cell MALT lymphoma is rare. Sjogren’s syndrome patients are at a higher risk of lymphoma, and therefore, a minor salivary gland biopsy should be recommended for patients diagnosed with a MALT lymphoma for a complete examination. Young patients can be successfully treated by surgery at the early stages of the disease. A follow-up done every six months is necessary for the timely detection of disease progression, and incorporation of multiple specialties is required.
